# Temporal and spatial patterns of recurrence in oral squamous cell carcinoma, a single-center retrospective cohort study in China

**DOI:** 10.1186/s12903-023-03204-7

**Published:** 2023-09-19

**Authors:** Yannan Wang, Tianru Yang, Chengwen Gan, Kai Wang, Bincan Sun, Mengxue Wang, Feiya Zhu

**Affiliations:** 1 Department of Oral and Maxillofacial Surgery, Center of Stomatology, Xiangya Hospital, Central South University, 87 Xiangya Road, Changsha, Hunan 410008 China; 2 Department of Vascular and Plastic Surgery, Guangdong Provincial People’s Hospital (Guangdong Academy of Medical Sciences), Southern Medical University, Guangzhou, China; 3 Research Center of Oral and Maxillofacial Tumor, Xiangya Hospital, Central South University, Changsha, China; 4 Institute of Oral Cancer and Precancerous Lesions, Central South University, Changsha, China; 5 Department of Oral and Maxillofacial Surgery, Hainan Provincial People’s Hospital, Haikou, Hainan China; 6 Department of Oral and Maxillofacial Surgery, The Second Xiangya Hospital of Central South University, Changsha, Hunan China

**Keywords:** Oral squamous cell carcinoma, Recurrence interval, Recurrence site, Early recurrence, Risk factor, Prognosis

## Abstract

**Background:**

Oral squamous cell carcinoma (OSCC) is an invasive cancer with a high recurrence rate. Most clinical studies have focused on the prognosis of patients with OSCC, few have investigated the causes and interventions that affect the recurrence. Our study is to explore the temporal and spatial patterns of recurrence in OSCC.

**Methods:**

234 OSCC patients with recurrence in our hospital and 64 OSCC patients with recurrence in TCGA database were included in the study. Log-rank test and Multivariate Cox Regression Analysis were used to determine whether there was a significant difference between each selected demographic or clinical factors and recurrence. The Kaplan–Meier method was used to plot survival curves for each recurrence interval.

**Results:**

The proportion of OSCC patients in clinical and TCGA with early recurrence was 93.6% and 84.4%, respectively. Age, chewing betel nut, previous radiotherapy, histopathological grading of the primary tumor (poorly differentiated), lymph node metastasis and postoperative infection were found to be associated with the timing of recurrence. It was found that tongue cancer has more regional recurrences, while buccal cancer is mostly local and loco-regional recurrences. The earlier the recurrence, the greater the possibility of local-regional recurrence and the worse the prognosis.

**Conclusion:**

Most of recurrent OSCC patients present early recurrence (< 18 months) with poor prognosis, and early recurrence is more prone to local recurrence. Moreover, recurrence site is related with primary site of OSCC.

## Background

Oral cancer is one of the most common malignancies, ranking the top 10 of all malignant tumors, with more than 450,000 new cases worldwide each year, accounting for 2.4% of all cancer deaths. More than 90% of oral cancers are oral squamous cell carcinoma (OSCC)[1]. The mainstays of OSCC treatment are surgery, chemotherapy, radiotherapy or a combination of these modalities. Unfortunately, despite the various efforts that have been made for OSCC [2, 3], the survival rate of OSCC has not remarkably increased in the last three decades, which remains below 60%[4].Recurrence (especially early recurrence) is one of the major causes of low survival rates of OSCC after definitive therapy. It has been reported that the cure rates of salvage surgery for patients with recurrence is 15%~67%, suggesting the role for surgical treatment after recurrence with both palliative and curative intent [5–9]. Therefore, regular and close follow-up examinations are particularly important for early diagnosis of recurrence and improvement of survival of OSCC patients [10]. At present, “one-size-fits all” follow up program cannot meet the clinical needs. First, this regimen may not be appropriate for some patients who may be at higher risk for a second event than others [11–13]. In addition, personalized follow-up schedule after primary tumor treatment has never been investigated [13]. While longer follow-up time and shorter follow-up interval may help us diagnose relapse early, they may also place an undue burden on patients, especially those in remote areas or with poor economic conditions.

Many literatures stated that the recurrence rate of OSCC varies from 7–47.4%[7, 14–18]. Despite the enormous impact of recurrence (local, loco-regional and regional) on the prognosis of OSCC, limited understanding is currently available about its frequency, exact location and development pattern [19–22]. Many clinicopathological factors and molecular markers are associated with recurrence of OSCC, including smoking, advanced clinical stage, poorly differentiated tumors, radiotherapy, microvascular invasion, and high positive lymph node ratio, etc. [7, 17, 19, 23–25]. However, the prognostic value of these clinicopathological factors is always uncertain and controversial [19, 25–27]. It is also worth noting that it’s not clear which clinicopathological factors and molecular biomarkers could identify the patients at high risk of early recurrence.

## Materials and methods

### Data collection methods

The patients’ data was collected and sorted from our hospital’s electronic database, which contains prior medical records and postoperative follow-up information.

### Study design and clinical samples

The study was approved by the Ethics Committee of Second Xiangya Hospital of Central South University. A total of 1560 consecutive OSCC patients underwent major surgery with primary reconstruction at our Department of Oral and Maxillofacial Surgery from January 2010 to December 2016. All patients underwent complete surgical resection with curative intent. For OSCC recurrence, 5 years is a cut-off point. If OSCC does not relapse in 5 years, the chance of recurrence will be greatly reduced. Therefore, the 5-year survival rate is always used to indicate the effect of OSCC treatment. Inclusion criteria were defined: (1) the patients were histologically confirmed with primary OSCC, (2) more than 5 years follow-up after surgical treatment without a loss or until death, (3) primary tumor without distant metastasis. Patients who had (1) a history of radiation or chemotherapy for oncological diseases of other origins and (2) inadequate clinical follow-up information were excluded.

Recurrence interval was defined as the duration from the end of initial treatment to the confirmation of recurrence by pathological examination after incisional biopsy. For patients with multiple tumor recurrences, only the time interval from the completion of the first treatment to the first recurrence was calculated. The recurrence interval is significantly associated with the survival rate of OSCC patients, with an interval < 18 months appearing to be a more dismal cut-off point for survival according to some literature reports [5, 9]. In this study, we used < 18 months as the cut-off point for defining early recurrence (ER).

To distinguish between recurrent tumors and new primary tumors, only patients with OSCC with the same or higher histologic grade as the primary tumor were included [5]. In this study, local recurrence, loco-regional recurrence and regional recurrence were all defined as recurrence. Recurrence arising only in the primary site was defined as local recurrence. Recurrence arising only in the neck was defined as regional recurrence. Recurrence arising in both the primary site and neck was loco-regional recurrence [28].

### TCGA database

TCGA (https://tcga-data.nci.nih.gov/tcga/) is a publicly available dataset. We screened 347 OSCC patients from the head and neck squamous cell carcinoma (HNSCC) dataset in TCGA and downloaded the clinical data for this study. Among them, 64 cases had recurrence.

### Treatment protocol and follow-up examinations

Treatment regimens for each patient in our department include resection of OSCC within the appropriate safe margin and the use of neck dissection recommended by Chinese guidelines [10, 29], which are mainly based on NCCN and ASCO, with no difference in treatment methods. Postoperative radiation was advised for cases with histologic evidence of lymph node metastasis, extracapsular extension of cervical lymph node metastasis and extensive local tumor burden. Typically, patients receive a follow-up examination every 1 month during the first year after completion of treatment, and a routine PET-CT is performed at the end of the first year. Follow-up examinations will be performed every 3 months in the second year, and then every 6 months in the third to fifth years. In case of clinical suspicion of local, loco-regional or regional recurrence, further re-staging procedures (CT, positron-emission CT, ultrasound, magnet-resonance imaging) may be performed as appropriate. Salvage surgery was considered the therapy for recurrent OSCC. No recurrence after 5 years of follow-up was considered to be the end of follow-up.

### Demographics and clinical data

Data obtained from medical records included demographic features, tumor sites, histopathology, grade, stage, total number of resected lymph nodes, total number of positive lymph nodes, total number of extracapsular infiltrations of lymph nodes, type of surgical procedure, TNM stage, type of salvage treatment, outcome, symptoms of patients with recurrence, time of symptom appearance, recurrence interval, diagnosis method of recurrence and location of recurrence, etc.

For comparison, all patients with recurrence were divided into 2 groups: early recurrence (ER) (n = 219) group and late recurrence (LR) (n = 15) group.

### Statistical analysis

Statistical analysis was performed using SPSS 25.0 software (SPSS Inc., USA), and R, version 3.6.1 (R Foundation for Statistical Computing, Vienna, Austria). The clinical prognostic risk factors associated with early recurrence were screened out by Log-rank test, and then these screened variables were further incorporated into the Multivariate Cox Regressive Analysis model for further analysis. The Kaplan–Meier method was used to plot survival curves for each recurrence interval. P value < 0.05 was considered statistically significant.

## Results

### The recurrence rate (RR) of OSCC patients was about 15%

From January 2010 to December 2016, a total of 1560 OSCC patients (1152 males and 408 females) with an average age of 49.62 ± 4.34 years who had undergone surgery were admitted to our hospital (Table 1). Among them, 234 patients (202 males and 32 females) relapsed within 5 years after surgery, with RR of about 15%, aged from 34 to 82 years, with an average age of 44 ± 7.23 years (See Table 2 for patient with detailed information). Further, we analyzed the clinical data of 347 patients with OSCC in TCGA database and found that the average age of the patients was about 61.58 ± 12.88 years, among which the youngest patient was 22 years old and the oldest was 88 years old. During the 5-year follow-up period, 64 patients developed recurrence, with RR of about 18.44%, and the average age of patients with recurrence was about 57.53 ± 14.45 years, including 22 female patients and 42 male patients.

**Table 1:** Clinical and demographic characteristics of the OSCC patients(n = 1560)

Variable			Patients’ numbers(n)
Gender		Male	1152
		Female	408
Age		＞60	856
		≤ 60	704
Smoking		Yes	624
		No	936
Drinking		Yes	583
		No	977
N classification		N0	764
		N1 ~ 3	796
T classification		1 ~ 2	862
		3 ~ 4	698
Stage		I-II	734
		III-IV	826
Grade		Well-moderation	712
		Poorly	848
Preoperative radiotherapy		Yes	134
		No	1406
		NA	20
Previous chemotherapy		Yes	33
		No	1327
		NA	200
Tumor recurrence		Yes	234
		No	1326
Neck dissection		Unilateral	1065
		Bilateral	395
		No	100
Postoperative infection		Yes	235
		No	1215
		NA	110
Tumor site		Tongue	792
		Buccal	397
		Gingiva	67
		Floor of mouth	76
		Jawbone	69
		Lips	35
		Palate	53
		Retromolar region	29
		Others*	42
Free flap repair		Yes	1245
		No	315

*Others include maxillary sinus, parotid gland, temporal bone, and neck skin

### More than 93.6% of OSCC patients with relapse had early recurrence

**Fig. 1:**
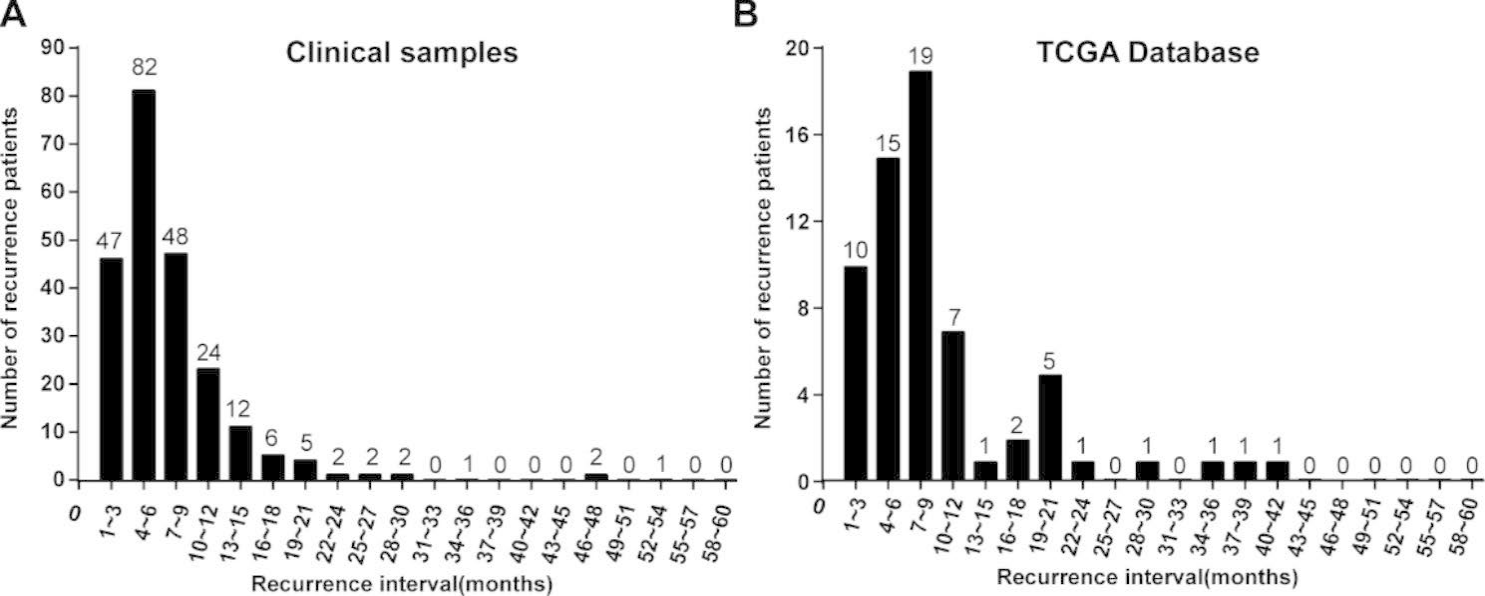
Recurrence interval in OSCC patients. **(A)** Of the relapsed patients from our department, 85.9% relapsed within 12 months, and 93.6% relapsed within 18 months. **(B)** Of the relapsed patients in TCGA database, 79.7% relapsed within 12 months, and 84.4% relapsed within 18 months

The number of recurrent patients at 3, 6, 12 and 18 months after surgery accounted for 20.1% (47/234), 55.1% (129/234), 85.9% (201/234), and 93.6% (219/234), respectively. Only 6.4% (15/234) of patients had recurrence within 18 to 60 months after surgery (Fig. 1A). After further analysis of the clinical data of 64 patients with recurrent OSCC from TCGA database, we found that the incidence of recurrence was 15.6% (10/64), 39.1% (25/64), 79.7% (51/64), and 84.4% (54/64) at 3, 6, 12, and 18 months after surgery, respectively. The incidence of recurrence was only 15.6% (10/64) after 18 months (Fig. 1B).

### Clinicopathological data in association with the timing of recurrence

To further evaluate the risk factors of early recurrence (ER) (recurrence interval < 18 months), we screened out 234 occurrence patients with complete data from 1560 patients. A significant correlation was found between the timing of recurrence and the age (P = 0.045), chewing betel nut (P = 0.048), previous radiotherapy (P = 0.038), histopathological grade of the primary tumor (poorly differentiated) (P = 0.048), lymph node metastasis (P = 0.032) and postoperative infection (P = 0.043) by Log-rank test (Table 2). However, in the Multivariate Cox Regression, histopathological grade(P = 0.870) is not a risk factor for ER of OSCC patients (Table 3).

**Table 2:** Statistical analyses of factors associated with ER in OSCC patients with Log-rank test

Variable	HR	HR.95 L- HR.95 H	*P*
Age(years) ≥ 60 VS＜60	1.341	1.098–2.058	0.045*
Gender Female VS Male	1.087	0.640–1.846	0.761
Chewing betel nut Yes VS No	1.481	1.140–2.221	0.048*
Drinking Yes VS No	0.816	0.560–1.189	0.290
Smoking Yes VS No	0.921	0.632–1.343	0.670
Previous radiotherapy Yes VS No	6.097	2.646–14.08	0.038*
Previous chemotherapy Yes VS No	0.990	0.502–1.957	0.981
Histopathological grade Well-moderate VS Poorly	0.872	0.612–0.997	0.048*
Lymph node metastasis Yes VS No	2.265	1.432–4.012	0.032*
Postoperative infection Yes VS No	1.650	1.051–3.324	0.043*
Stage I-II VS III-IV	0.921	0.609–1.392	0.700
T classification 1–2 VS 3–4	0.866	0.551–1.340	0.530
Free flap repair Yes VS No	1.043	0.903–1.101	0.212
Neck dissection Unilateral VS Bilateral	1.182	0.551–2.547	0.120

**Table 3:** Statistical analyses of factors associated with ER in OSCC patients with Multivariate Cox Regression Analysis

Variable	HR	HR.95 L- HR.95 H	*P*
Age(years) ≥ 60 VS＜60	1.89	1.14–3.12	0.047*
Chewing betel nut Yes VS No	2.96	1.88–4.66	0.041*
Previous radiotherapy Yes VS No	3.70	1.12–12.22	0.032*
Histopathological grade Well-moderate VS Poorly	1.05	0.58–1.89	0.870
Lymph node metastasis Yes VS No	2.48	1.47–3.99	0.021*
Postoperative infection Yes VS No	2.58	1.60–4.32	0.035*

### Tongue cancer is mostly regional recurrences, while buccal cancer is mostly local recurrences

Among all the 234 patients with postoperative recurrence in clinical, 105 patients had local recurrence, 102 patients had regional recurrence, and 27 patients had loco-regional recurrence. Similarly, of all the 64 patients with recurrence in TCGA, 25 patients had local recurrence, 24 patients had regional recurrence, and 15 patients had loco-regional recurrence. The ratio of local recurrence to regional recurrence was about 1.03:1 (105:102) in clinical, which was similar to the ratio of 1.04:1(25:24) in TCGA database. The ratio of local recurrence to loco-regional recurrence was about 3.89:1 (105:27) clinically and 1.67:1(25:15) in TCGA database.

**Fig. 2:**
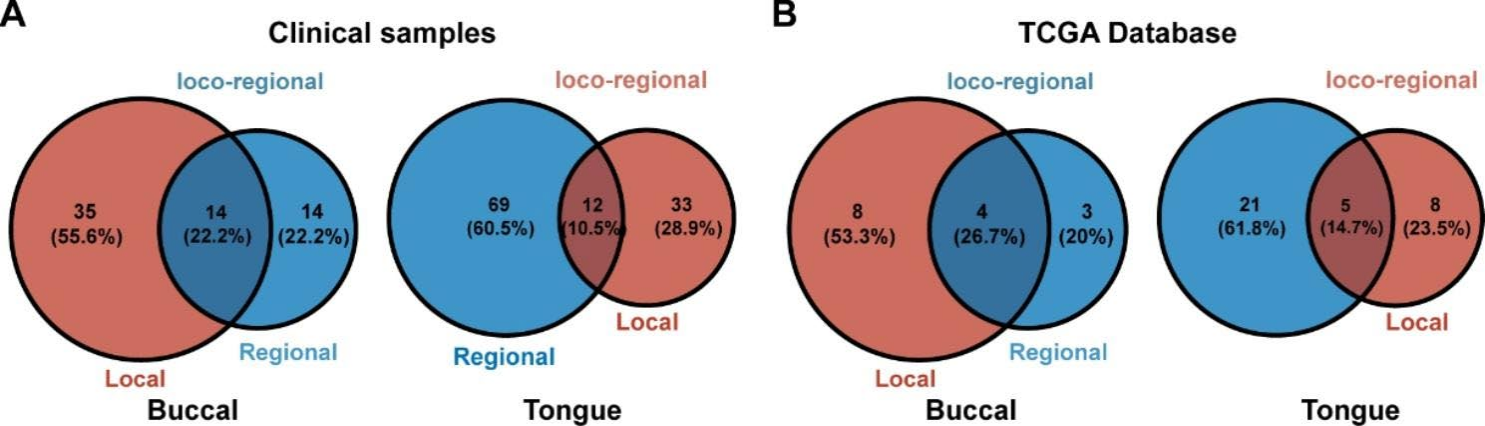
The composition of recurrent types of buccal cancer and tongue cancer. **(A)** The results of clinical data showed that the proportions of local, local-regional and regional recurrence were 55.6%, 22.2% and 22.2% in patients with buccal cancer recurrence, respectively. The proportions of local, local-regional and regional recurrence in patients with recurrent tongue cancer were 28.9%, 10.5% and 60.5%, respectively. **(B)** TCGA data showed that among patients with buccal cancer recurrence, the proportions of local, local-regional and regional recurrence were 53.3%, 26.7% and 20%, respectively. The proportion of local, local-regional and regional recurrence in patients with recurrent tongue cancer were 23.5%, 14.7% and 61.8%, respectively

After further analysis of the different sites of OSCC recurrence, it was found that among the 63 patients with buccal cancer recurrence, 35 patients had local recurrence alone, 14 patients had regional recurrence, and 14 patients had loco-regional (Fig. 2A). Of the 114 cases of tongue cancer recurrence, 33 cases had local recurrence, 69 cases had regional recurrence, and 12 cases had loco-regional recurrence (Fig. 2B). Combined with the data of 64 patients with recurrent OSCC in TCGA database, and through cross tabulation with chi-squared testing, we found that buccal cancer is more prone to local recurrence (Table 4), while tongue cancer is more prone to regional recurrence (Table 5). It is worth noting that our clinical results showed that buccal cancer was more prone to loco-regional recurrence than tongue cancer (*P* = 0.035), but the results from TCGA database showed there was no statistical difference in the probability of loco-regional recurrence between them (*P* > 0.05) (Table 6).

**Table 4:** The relationship between the location of primary tumor and local recurrence in OSCC patients

Variable	Tumor site	Local recurrence		*P*
		Yes	No	
Clinical Samples				
	Tongue	33(28.9%)	81(71.1%)	< 0.001***
	Buccal	35(55.6%)	28(44.4%)	
TCGA Database				
	Tongue	8(23.5%)	26(76.5%)	0.04*
	Buccal	8(53.3%)	7(46.7%)	

**Table 5:** The relationship between the location of primary tumor and regional recurrence in OSCC patients

Variable	Tumor site	Regional recurrence		*P*
		Yes	No	
Clinical Samples				
	Tongue	69(60.5%)	45(39.5%)	< 0.0001****
	Buccal	14(22.2%)	49(77.8%)	
TCGA Database				
	Tongue	21(61.8%)	13(38.2%)	0.007**
	Buccal	3(20%)	12(80%)	

**Table 6:** The relationship between the location of primary tumor and loco-regional recurrence in OSCC patients

Variable	Tumor site	Loco-regional recurrence		*P*
		Yes	No	
Clinical Samples				
	Tongue	12(10.5%)	102(89.5%)	0.035*
	Buccal	14(22.2%)	49(77.8%)	
TCGA Database				
	Tongue	5(14.7%)	29(85.3%)	0.32
	Buccal	4(26.7%)	11(73.3%)	

### The earlier the recurrence, the greater the possibility of local-regional recurrence and the worse the prognosis

**Fig. 3:**
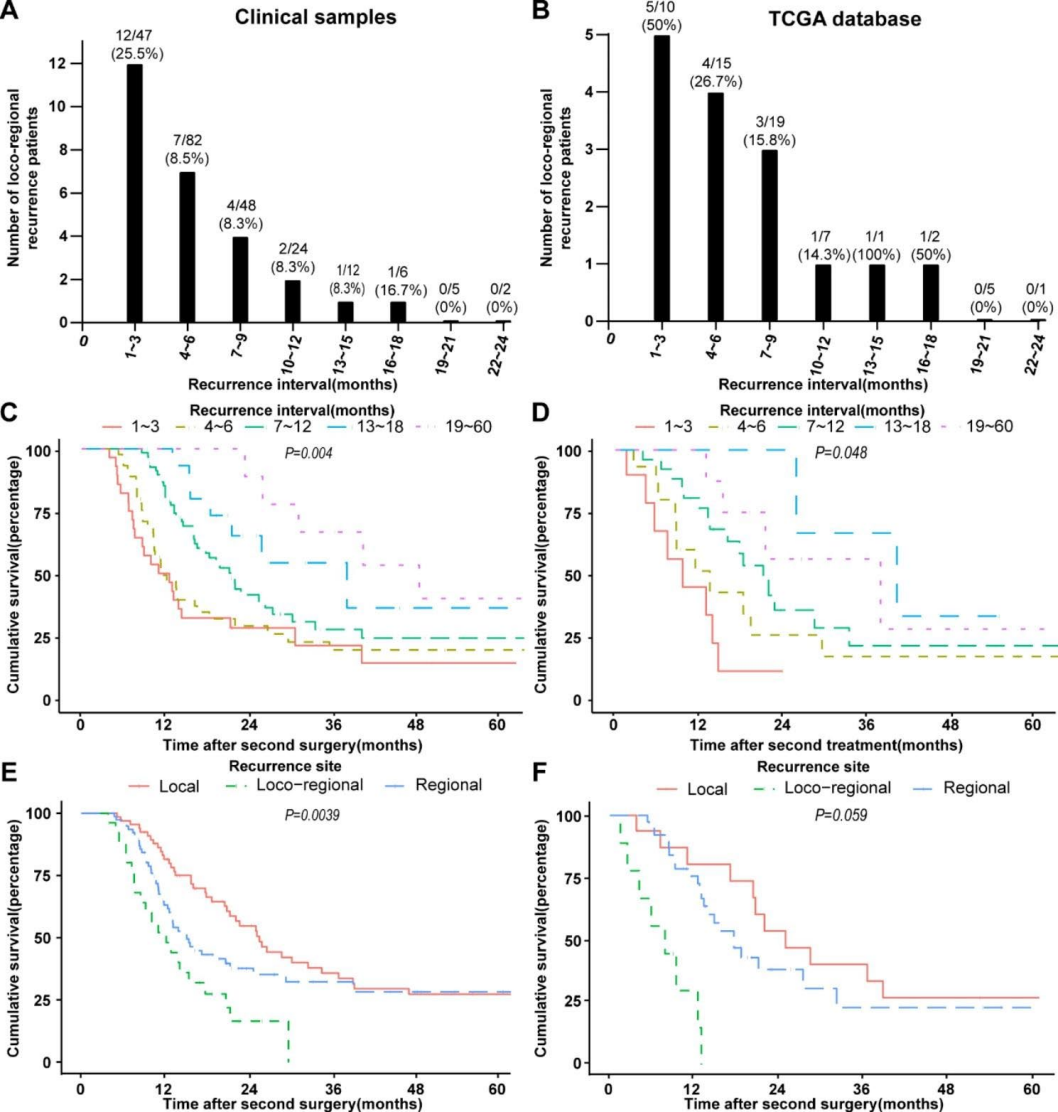
The relationship between recurrence interval and local-regional recurrence and prognosis. Among patients with local-regional recurrence, the number and ration of cases within 1 ~ 3, 4 ~ 6, 7 ~ 9, 10 ~ 12, 13 ~ 15, 16 ~ 18 months **(A)** from clinical was 12(25.5%), 7(8.5%), 4(8.3%), 2(8.3%), 1(8.3%), 1(16.7%), respectively. **(B)** From the TCGA database, the number (and ration) were 5(50%), 4(26.7%), 3(15.8%), 1(14.3%), 1(100%), 1(50%), respectively. (Ration = The number of loco-regional recurrence patients/The number of recurrence patients). The 5-year survival rate of OSCC patients with recurrence in **(C)** clinical and **(D)** TCGA database after the second surgery. The 5-year survival rate of OSCC patients with local, loco-regional and regional recurrence from **(E)** 170 OSCC patients in our hospital and **(F)** TCGA database

After analyzing the relationship between recurrence time and recurrence region of OSCC patients with recurrence in clinical and TCGA databases, we found that among patients with local-regional recurrence, the number (and ration) of cases within 1 ~ 3, 4 ~ 6, 7 ~ 9, 10 ~ 12, 13 ~ 15, 16 ~ 18 months from clinical was 12(25.5%), 7(8.5%), 4(8.3%), 2(8.3%), 1(8.3%), 1(8.3%), respectively (Fig. 3A). According to TCGA database, the number (and ration) was 5(50%), 4(26.7%), 3(15.8%), 1(14.3%), 1(100%), 1(50%), respectively (Fig. 3B). These results showed that the earlier the recurrence, the more likely it was to be loco-regional recurrence. Further, a survival analysis of 170 OSCC patients who underwent secondary surgery after recurrence showed that the earlier the recurrence, the worse the prognosis (Fig. 3C). The result was consistent with that of 63 patients who received secondary treatment in TCGA database (Fig. 3D). Meanwhile, it was also found that among 170 OSCC patients, the patients of loco-regional recurrence had the lowest 5-year survival rate after surgery, while local recurrence had a relatively high 5-year survival rate (Fig. 3E). However, the results from TCGA database showed there was no statistically difference (P = 0.059, Fig. 3F), which may be due to the small sample size of TCGA.

## Discussion

Tumor recurrence is associated with deterioration in patients outcomes and is therefore one of the major issues for OSCC treatment [30]. Survival rates of early relapse were reported to be much lower than late relapse [31]. Meanwhile, some literatures showed that the survival rate of patients with OSCC recurrence is related to the site of recurrence [21, 23, 25, 32, 33]. However, current medical literatures contain few studies on temporal and spatial patterns of OSCC recurrence.

Due to the global imbalance of medical care and the continuous improvement of OSCC diagnosis and treatment, previous scholars’ reports on RR were not consistent, which was ranging from 7–47.4%[5, 7, 14–18]. In this study, the 5-year follow-up data of 234 OSCC patients form our department and 64 OSCC patients from TCGA database were analyzed, the results indicated that the 5-year recurrence rates of OSCC patients in clinical and TCGA were 15% (234/1560) and 18.44% (64/347), respectively. The research of Rogers SN [34] and Vázquez-Mahía I [35] have shown that up to 86% of patients with OSCC relapsed within 2 years after the first treatment, and that those who relapsed early had a worse prognosis than those who relapsed afterwards [18, 34–38]. In our hospital, 93.6% of the recurrences occurred within 18 months (Fig. 1A), which was significantly higher than 84.4% in TCGA database (Fig. 1B) or 60% reported by Annelies Weckxa [18]. We speculated that this difference may be related to the betel nut chewing habits of East Asian. In this regard, we performed Log-rank test on the clinicopathological factors of early and late recurrence of OSCC patients and found that chewing betel nut was associated with the timing of recurrence (Table 2). After further subdividing the recurrence interval, we found that the recurrent patients at 3, 6, 12 and 18 months after surgery accounted for 20.1%, 55.1%, 85.9%, and 93.6%, respectively. Only 6.4% of patients relapsed within 18 ~ 60 months after surgery (Fig. 1A). Similar results were obtained from TCGA database. 79.7% of relapses occurred within 12 months after the first treatment, and 84.4% of relapsed patients relapsed early (Fig. 1B). Therefore, given that nearly 90% of recurrence occurred within 18 months after surgery (approximately 80% of which occurred within 1 year after surgery), and that close follow-up and early diagnosis could improve the success rate of rescue for relapsed patients.

We believe that regular examinations should be performed within 24 months after surgery, while continuing follow up medical history and physical examinations for about 5 years based on individual risk assessment. In our hospital, it is recommended to pay regular examination every month for 1 year after the operation and keep checkups every 3 months from 1 to 2 years after the operation. Superficial recurrence can be diagnosed by pathological biopsy, while deep recurrence can only be diagnosed based on the patient’s symptoms (such as local pain, etc.), signs and imaging examinations [18, 39]. Through this study, we found that tongue cancer is more prone to regional metastasis, while buccal cancer is more likely to recur locally. At the same time, age, betel nut chewing, previous radiotherapy history, lymph node metastasis and postoperative infection are risk factors for early recurrence of OSCC. Combining with the studies of previous scholars [40–42], we believe that MRI/CT/PET-CT examination, especially for patients with deep neck pain, MRI/CT examination or even PET-CT should be performed immediately to confirm the diagnosis. Considering that buccal cancer tends to recur locally [39, 42], we believe that for buccal cancer patients, the definite diagnosis can be confirmed by combining the patient’s signs, visual inspection, palpation and pathological biopsy. Additionally, NCCN guidelines suggest that there may be little proven benefit in further imaging if the initial 3-month FDG-PET/CT scan is negative. If FDG-PET/CT at 3 months post-surgery is negative, then there are no data to support a substantial benefit for further imaging in an asymptomatic patient [43]. However, similar to the study results of Chonji Fukumoto [40], considering that the probability of recurrence in patients still exists after 18 months after surgery, although the proportion is already very low, we routinely recommend patients undergo PET-CT examination at 12 months after surgery to rule out early microscopic lesions.

Further, we analyzed the clinicopathological factors related to early and late recurrence. Abundant evidence exists for association between the occurrence interval and grade of primary tumor [18, 19, 25, 37], lymph node metastasis [18, 21, 23] and previous radiotherapy [44, 45]. Tumor pathologic features such as DOI (depth of invasion), PNI(perineural invasion), LVI (lymphovascular invasion), and ENE (extranodal extension) ,etc. have been shown to be associated with lymph node metastasis in OSCC [46]. In this study, we have not done an in-depth analysis of these factors, which would require more data and a more detailed analysis. Previously clinical studies have shown that young age [47, 48], postoperative infection [49, 50] are related to the recurrence in many tumors. Similarly, chewing betel nut has been shown to be related to the progression of OSCC in multiple studies [51, 52]. In this study, a significant correlation was found by Log-rank test between the recurrence interval and age (P = 0.045), chewing betel nut (P = 0.048), previous radiotherapy(P = 0.038), lymph node metastasis (P = 0.032), and postoperative infection (P = 0.043) (Table 2). The study-is to our best knowledge-the first to show a significant association between the recurrence interval and age, chewing betel nut and postoperative infection.

In addition to the recurrence interval, some other clinical factors are found to be associated with the recurrence site. As described in previously studies, OSCC patients with primary lymph node metastases to the neck have an increased recurrence rate [28, 53–55]. Late pN status, extracapsular spread, perineural infiltration, vascular/lymphatic embolism, diffuse infiltration and neck dissection were found to be associated with regional (neck) recurrence which was an important prognostic factor for overall survival (OS)[56]. While poor differentiation, location (hard palate and retromolar trigone), bone invasion, lymphatic invasion, surgical margins and invasion depths were significantly associated with local-regional recurrence [57]. All of these risk factors lead to poorer tumor control.

Although there have been numerous studies on the incidence of local, regional, and local-regional recurrence in patients with OSCC, fewer have been reported on the proportion of these three in the same group of relapsed patients or in different OSCC subtypes. Studies by Yasmine Ghantous [58] and Thomas Mücke [59] found that the probability of local recurrence in OSCC was about 64.9%[58, 59], which was more common than loco-regional recurrence, and the ration was 2.58:1(64.9%:25.1%) [59]. While the study of Troeltzsch showed that regional recurrence is usually combined with local recurrence within 2 years of the initial diagnosis of OSCC [60].Through our study, we found that among all the OSCC patients with postoperative recurrence in clinical (234 patients) and TCGA (64 patients), the ratios of local recurrence to loco-regional recurrence were about 3.89:1 (105:27) and 1.67:1(25:15), respectively. These results were consistent with the study of Thomas Mücke [59]. Further, we found that the ratio of local recurrence to regional recurrence was about 1.03:1 (105:102) in clinical, which was similar to the ratio of 1.04:1(25:24) in TCGA database. Furthermore, based on the location of the primary tumor, we analyzed the spatial pattern of recurrence of OSCC in different locations. Through cross tabulation with chi-squared testing, we were surprised to find that buccal cancer was more prone to local recurrence (Table 4), while tongue cancer was more prone to regional recurrence (Table 5). The reasons may be due to the local anatomy and the location of lymphatic drainage [10, 61]. Unlike tongue cancer, buccal cancer has been shown to develop in an orderly progression, skip metastasis was rare [62], however, its specific biological mechanism is still unclear and needs further study. It is worth noting that our results of clinical samples showed that buccal cancer was more prone to regional recurrence (P = 0.035) but results of TCGA database showed no statistical difference in the probability of regional recurrence between the two groups (P > 0.05) (Table 6). Differences in ethnicity, number of cases, and dietary habits (for example, most Chinese OSCC patients chew betel nut while Americans do not) could contribute to the above difference.

More and more studies have shown that the recurrence interval and recurrence location were related to the prognosis of OSCC patients [5, 9, 18, 20, 37]. However, there are currently few studies on the relationship between the recurrence interval and the recurrence site. In this study, we found that the earlier the recurrence, the more likely it is to be loco-regional recurrence (Fig. 3A and B). Further, a survival analysis of patients who underwent secondary surgery after recurrence showed that the earlier the recurrence, the lower the survival rate (Fig. 3C, 3D). These results indicated that patients with local-regional recurrence have a wide range of second tumors, rapid tumor progression, and poor prognosis. The cause has nothing to do with the complete resection of the primary tumor, may be related to tumor heterogeneity [63, 64]. Considering the postoperative quality of life and survival rate, we should be cautious about reoperation for such patients.

## Conclusion

In the study, we found that not only recurrence interval, but also recurrence site was associated with the OS of OSCC.

Considering that most patients relapse within 18 months(nearly 90%), it is recommended to pay regular examination every month for 1year after the operation. Follow-up examinations will be performed every 3 months in the second year, and then every 6 months in the third to fifth years. For the surveillance of patients with OSCC after primary surgery, we believe that MRI/CT/PET-CT examination, especially for patients with deep neck pain, MRI/CT examination or even PET-CT should be performed immediately to confirm the diagnosis. At 1 year after surgery, it is also desirable to perform PET-CT to rule out early microscopic lesions. In addition, considering the poor prognosis of patients with earlier recurrence, re-operation should be cautious.

## Data Availability

All the data in the manuscript are available from the corresponding author on reasonable request.
